# Association of circulating SLAMF7^+^Tfh1 cells with IgG4 levels in patients with IgG4-related disease

**DOI:** 10.1186/s12865-020-00361-0

**Published:** 2020-06-01

**Authors:** Kazuhiko Higashioka, Yuri Ota, Takashi Maehara, Masafumi Moriyama, Masahiro Ayano, Hiroki Mitoma, Mitsuteru Akahoshi, Yojiro Arinobu, Takahiko Horiuchi, Seiji Nakamura, Koichi Akashi, Hiroaki Niiro

**Affiliations:** 1 Department of Medicine and Biosystemic Science, Graduate School of Medical Sciences, Kyushu University, 3-1-1 Maidashi Higashi-ku, Fukuoka, 812-8582 Japan; 2 Section of Oral and Maxillofacial Oncology, Division of Maxillofacial Diagnostic and Surgical Sciences, Faculty of Dental Science, Kyushu University, 3-1-1 Maidashi Higashi-ku, Fukuoka, 812-8582 Japan; 3 Department of Internal Medicine, Kyushu University Beppu Hospital, 4546 Tsurumihara, Beppu, Oita 874-0838 Japan; 4 Department of Medical Education, Faculty of Medical Sciences, Kyushu University, 3-1-1 Maidashi Higashi-ku, Fukuoka, 812-8582 Japan

**Keywords:** SLAMF7, IgG4-RD, Follicular helper T cells, IL-21, IL-10, B cells

## Abstract

**Background:**

Follicular helper CD4^+^ T (Tfh) cells have a critical role in IgG4 production by B cells in IgG4-related disease (IgG4-RD). Recent studies including ours showed that SLAMF7^+^CD4^+^ T cells are an important pathological driver of IgG4-RD. In this study, we have sought to elucidate a relationship between helper CD4^+^ T (Th), particularly Tfh, cells and SLAMF7^+^ CD4^+^ T cells in IgG4-RD.

**Results:**

The patients with IgG4-RD enrolled in this study were aged 66 ± 12 years and their titers of serum IgG4 were 372 ± 336 mg/dl. Th1 cells, activated circulating Tfh1 (cTfh1), and activated cTfh2 cells increased in IgG4-RD. SLAMF7 was mainly expressed on Th1 and cTfh1, but not cTfh2, cells in the patients. SLAMF7^+^ cTfh1 cells were PD-1/CD28 double-positive, whereas SLAMF7^+^ Th1 cells were CD28 negative. Positive correlations were noted between serum IgG4 levels and the number of activated cTfh2 cells and SLAMF7^+^ cTfh1 cells, but not SLAMF7^+^ Th1 cells. Intriguingly, among cTfh1 cells, activated SLAMF7^+^ cTfh1 cells were high producers of IL-10 along with IL-21. Blimp-1, but not Bcl-6, mRNA was expressed at high levels in activated SLAMF7^+^ cTfh1 cells. In addition to CD4^+^ T cells, the frequency of SLAMF7^+^ fraction was higher in memory B cells than naïve B cells in patients with IgG4RD. Finally, upon stimulation via B-cell receptor and CD40, Tfh1-associated cytokines, IL-21 and IFN-γ, most significantly induced SLAMF7 expression in memory B cells.

**Conclusions:**

Together, these results suggest that circulating SLAMF7^+^ Tfh1 cells, along with Tfh2 cells, play a pathologic role in IgG4 production in IgG4-RD.

## Background

IgG4-related disease (IgG4-RD) is a novel clinical entity characterized by tumefactive lesions, lymphoplasmacytic infiltrates consisting of CD4^+^ T cells and numerous IgG4^+^ plasma cells, and tissue fibrosis [1, 2]. Tertiary germinal center (GC) formation is frequently observed in diseased tissues [3]. The underlying pathogenesis of this disease, however, remains poorly understood.

CD4^+^T cell subsets are classified depending on the patterns of cytokine production, namely T helper 1 (Th1), T helper 2 (Th2), T helper 17 (Th17) and T follicular helper (Tfh) cells. Tfh cells (CXCR5^+^CD4^+^) mainly reside in the GC and produce IL-21 that helps the generation of memory B cells and plasma cells [4]. Of note, humans have peripheral blood (PB) counterpart of Tfh cells termed circulating Tfh (cTfh). cTfh cells can be further divided into three subsets by additional surface markers, namely cTfh1, cTfh2, and cTfh17 [5]. Recent studies highlight a pathogenic role of Tfh cells in IgG4-RD [3, 6–10].

Kubo et al. showed that proportion of cTfh cells correlates with that of plasmablasts in IgG4-RD [6]. In terms of cTfh subsets, however, there are some discrepancies between these studies. Akiyama et al. showed that cTfh2, but not cTfh1, cells are critical for serum IgG4 levels in IgG4-RD [7, 8]. On the other hand, Chen et al. showed that cTfh1 as well as cTfh2 cells in IgG4-RD efficiently promote plasma cell differentiation [9]. Thus, a pathogenic role of each Tfh subset in IgG4 production remains somewhat elusive.

SLAMF7 is a member of SLAM (Signaling Lymphocyte Activation Molecule) family receptors expressed on various cells including NK cells, CD8^+^ T cell subsets, and activated B cells [11–13]. Recent studies including ours showed that SLAMF7^+^ CD4^+^ cytotoxic T cells (CTL) are a critical pathologic driver in IgG4-RD [14, 15]. Intriguingly, B cell depletion reduces the number of SLAMF7^+^ CD4^+^ CTLs and disease activity [14], underscoring the close interaction between B cells and SLAMF7^+^ CD4^+^ CTLs.

To elucidate a relationship between Tfh cells and SLAMF7^+^ CD4^+^ CTLs in IgG4 production by B cells in IgG4-RD, we examined the phenotype of CD4^+^ T cells and SLAMF7^+^ subsets using flow cytometry. Moreover, the correlation of the number of CD4^+^ T cell subsets with serum IgG4 levels as well as cytokine production of SLAMF7^+^ subsets in IgG4-RD was assessed. We also examined the expression levels of SLAMF7 in naïve and memory B cells in patients with IgG4-RD and further investigated the underlying mechanism of how SLAMF7 expression is induced in memory B cells.

## Results

### Th1 cells, activated cTfh1, and activated cTfh2 cells increase in IgG4-RD

**Fig. 1:**
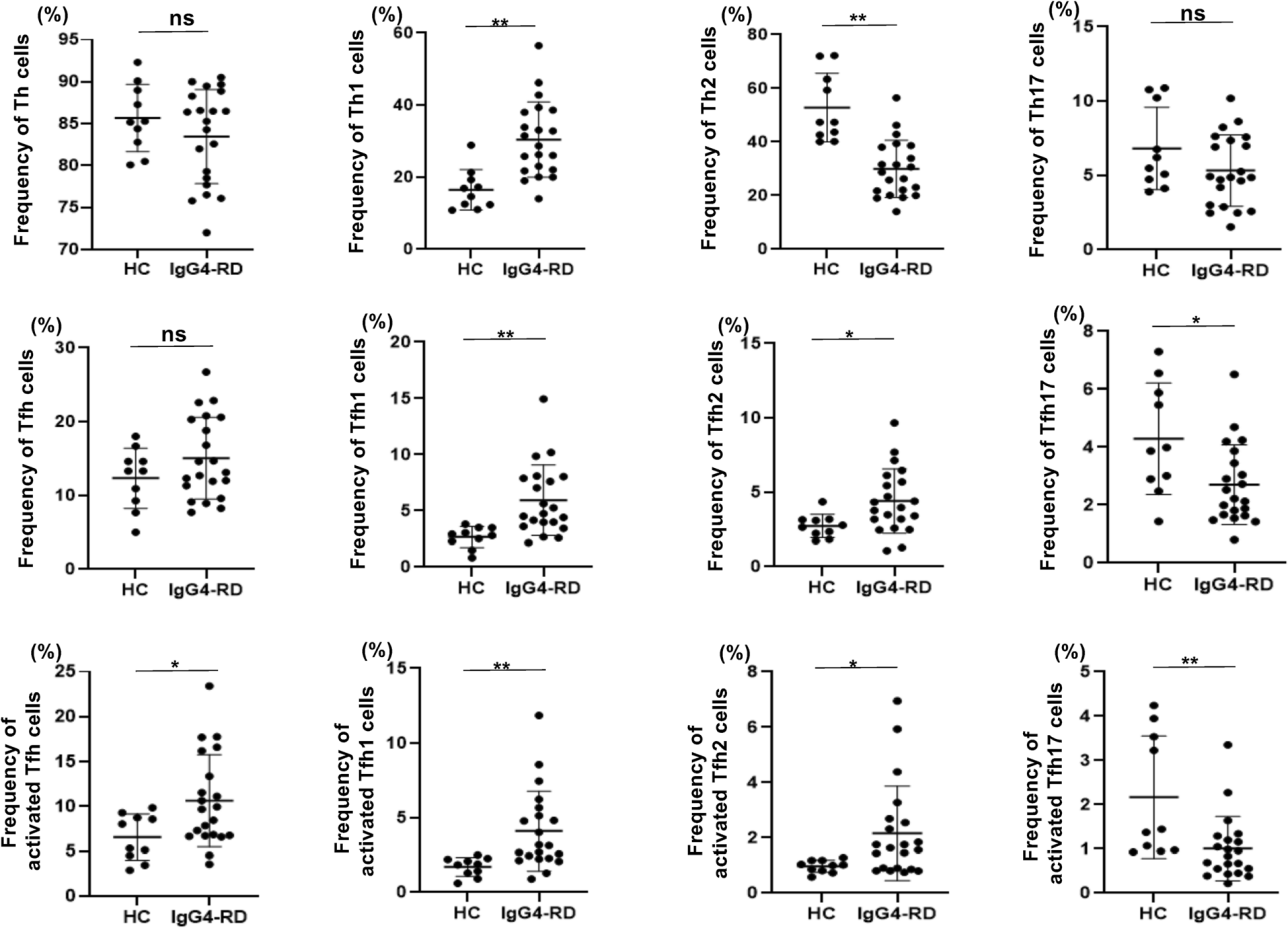
Phenotype of each CD4^+^ T cell subset in HCs and patients with IgG4-RD. Frequency of each CD4^+^ T cell subset in the peripheral blood of HCs and patients with IgG4-RD. Th cells are defined as CD3^+^CD4^+^CXCR5^−^, Th1 cells as CD3^+^CD4^+^CXCR5^−^CXCR3^+^CCR6^−^, Th2 cells as CD3^+^CD4^+^CXCR5^−^CXCR3^−^CCR6^−^, Th17 cells as CD3^+^CD4^+^CXCR5^−^CXCR3^−^CCR6^+^, Tfh cells as CD3^+^CD4^+^CXCR5^+^, Tfh1 cells as CD3^+^CD4^+^CXCR5^+^CXCR3^+^CCR6^−^, Tfh2 as CD3^+^CD4^+^CXCR5^+^CXCR3^−^CCR6^−^, Tfh17 cells as CD3^+^CD4^+^CXCR5^+^CXCR3^−^CCR6^+^, activated Tfh cells as PD-1^+^ fraction of each Tfh subset. * indicates *p* < 0.05, ** indicates *p* < 0.01, ns: non-significant

We first determined the frequency of CD4^+^ T cell subsets including Th1, Th2, Th17, cTfh1, cTfh2, and cTfh17 in HCs and IgG4-RD patients. The frequency of activated fractions of Tfh cells was also determined. The percentage of Th1 cells, cTfh1 cells, cTfh2 cells, activated cTfh1 cells, and activated cTfh2 cells was significantly increased in IgG4-RD patients as compared with that in HCs (30.4 ± 10.2% vs 16.5 ± 5.34%, *p* < 0.001; 5.94 ± 3.06% vs 2.67 ± 0.88%, *p* = 0.002; 4.41 ± 2.11% vs 2.73 ± 0.73%, *p* = 0.018; 4.10 ± 2.62% vs 1.70 ± 0.59%, *p* = 0.003; 2.16 ± 1.67% vs 0.97 ± 0.21%, *p* = 0.045), whereas that of Th2, cTfh17, and activated cTfh17 cells was significantly decreased (29.9 ± 10.4% vs 52.8 ± 12.1%, *p* < 0.001; 2.70 ± 1.35% vs 4.29 ± 1.83%, *p* = 0.02; 1.00 ± 0.71% vs 2.17 ± 1.32%, *p* = 0.005) (Fig. 1). These results suggest that Th1 cells, activated cTfh1, and cTfh2 cells are involved in the pathogenesis of IgG4-RD.

### Th1 cells and cTfh1 cells express SLAMF7 in IgG4-RD

**Fig. 2:**
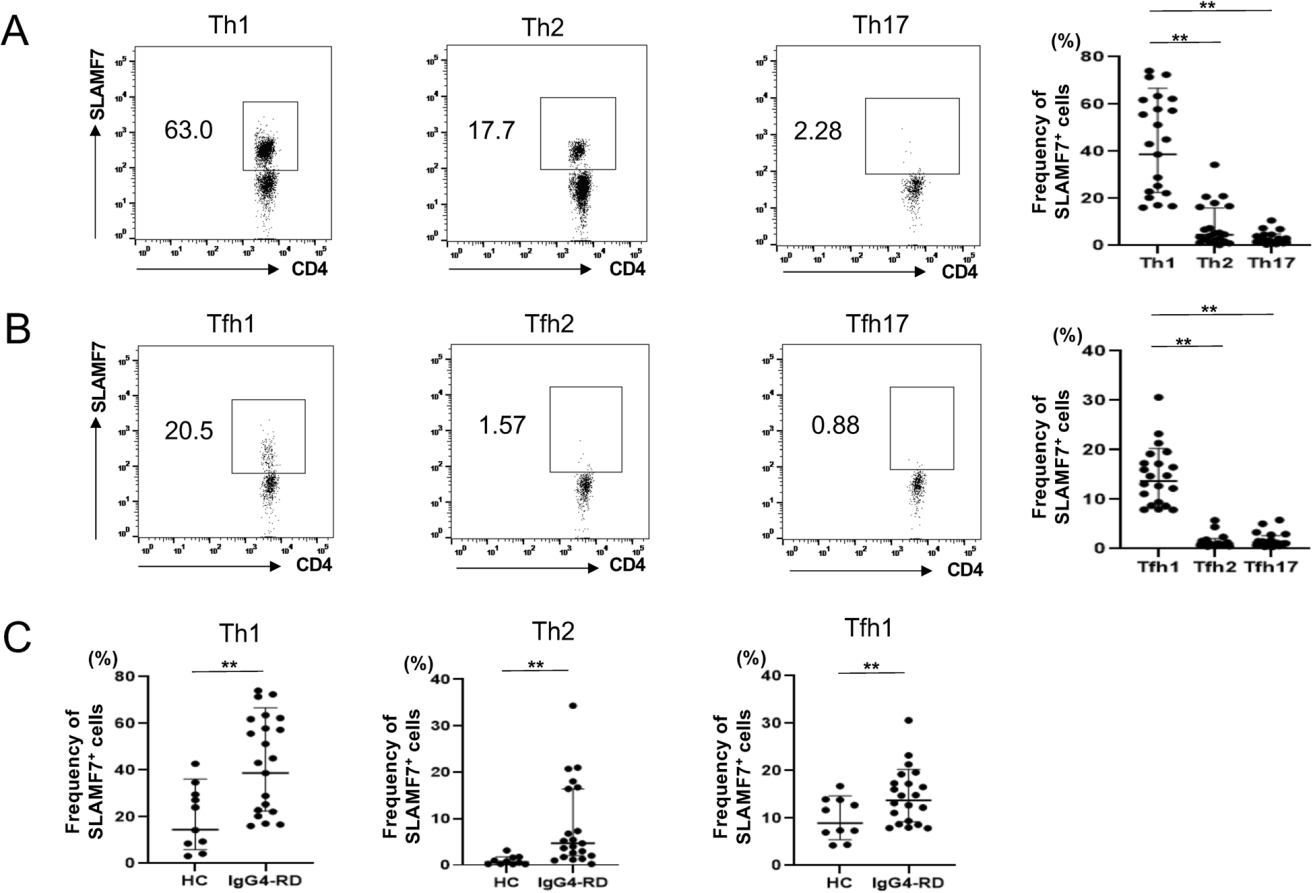
SLAMF7 expression in Th and Tfh subsets in patients with IgG4-RD. Comparison of the frequency of SLAMF7^+^ fraction of each Th subset (**a**) and Tfh subset (**b**) in patients with IgG4-RD patients. **c** Comparison of the frequency of SLAMF7^+^ fraction of Th1, Th2 and Tfh1 subsets in HCs and patients with IgG4-RD. **indicates *p* < 0.01

Recent studies including ours showed that SLAMF7^+^ CD4^+^ cytotoxic T cells (CTL) are a critical pathologic driver in IgG4-RD [14, 15]. We next checked the expression of SLAMF7 on each cTfh and Th subset. As shown in Fig. 2a, the percentage of SLAMF7^+^ cells in Th1 population was significantly higher than that in Th2 and Th17 populations in IgG4-RD patients (44.0 ± 20.0% vs 8.44 ± 8.88% vs 2.99 ± 2.54%, *p* < 0.001). On the other hand, among Tfh cells, SLAMF7^+^ cells were almost exclusively confined to cTfh1 subsets as compared with cTfh2 and cTfh17 subsets in IgG4-RD patients (Fig. 2b: 14.8 ± 5.75% vs 1.38 ± 1.30% vs 1.68 ± 1.47%, *p* < 0.001). Notably, the proportion of SLAMF7^+^ cells in Th1 and cTfh1 cells from IgG4-RD patients was significantly higher than that from HCs (Fig. 2c). Th2 cells also express higher levels of SLAMF7 expression, but to a lesser extent, than Th1 cells in IgG4-RD patients. These results suggest that SLAMF7 was mainly expressed on Th1 and cTfh1 cells in IgG4-RD patients.

### Expression of PD-1 and CD28 in SLAMF7^+^ fraction of Th1 and cTfh1 cells

**Fig. 3:**
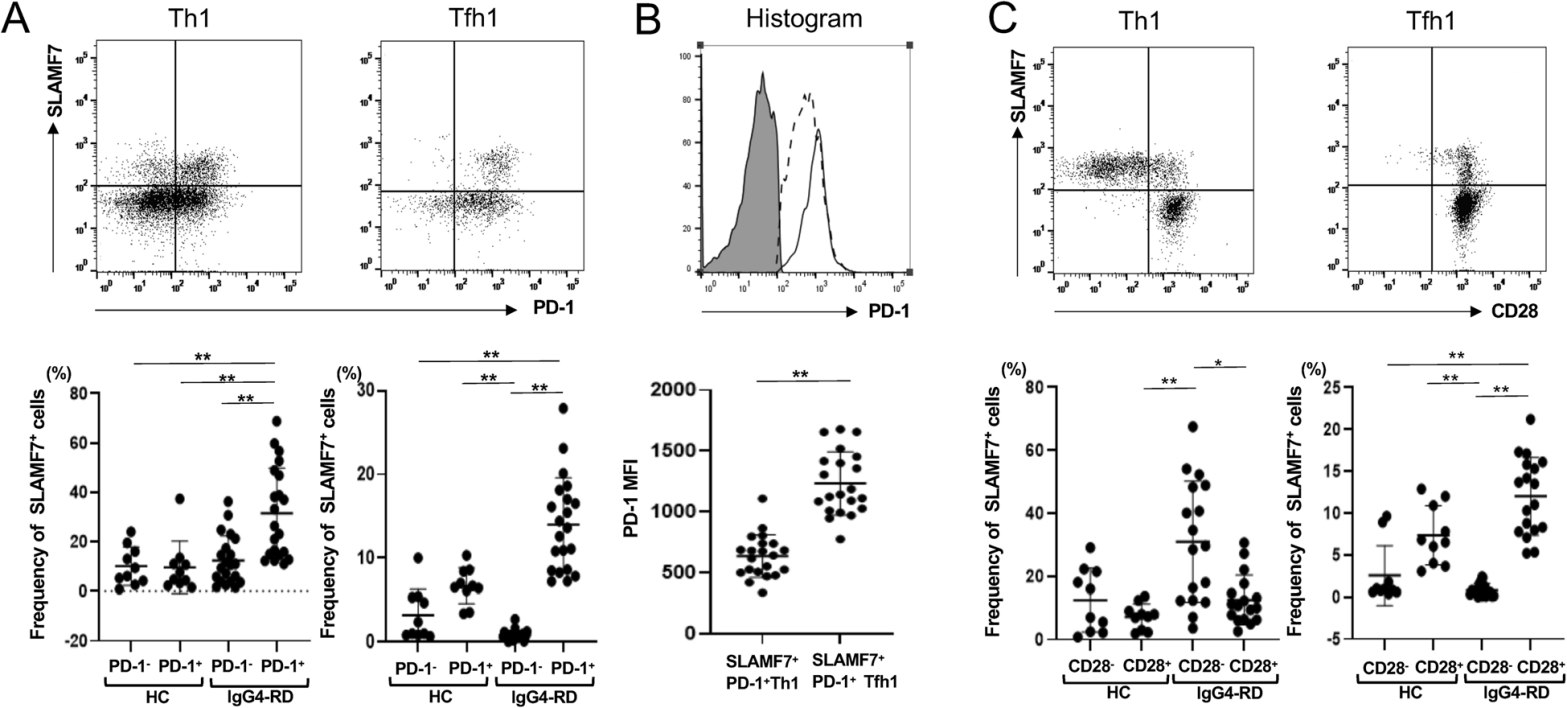
Expression levels of PD-1 and CD28 in SLAMF7^+^ Tfh1 and Th1 cells in patients with IgG4-RD. **a** Comparison of the frequency of SLAMF7^+^ fraction of Th1 and Tfh1 cells in HCs and patients with IgG4-RD in the absence or presence of PD-1. **b** Comparison of PD-1 expression levels on Th1 and Tfh1 cells in IgG4-RD patients (shadow: isotype control, dotted line: PD-1 in SLAMF7^+^PD-1^+^Th1 cells, solid line: PD-1 in SLAMF7^+^PD-1^+^Tfh1 cells). **c** Comparison of the frequency of SLAMF7^+^ fraction of Th1 and Tfh1 cells in HCs and patients with IgG4-RD in the absence or presence of CD28. * indicates *p* < 0.05, ** indicates *p* < 0.01

Given that Th1 and cTfh1 cells constitute heterogeneous populations, we determined the expression of PD-1 and CD28 in SLAMF7^+^ fraction of Th1 and cTfh1 cells in HCs and IgG4-RD patients. As shown in Fig. 3a, the frequency of SLAMF7^+^ population was significantly higher in PD-1^+^ than PD-1^−^ Th1 cells in IgG4-RD patients (30.8 ± 18.0% vs 12.7 ± 10.3%, *p* < 0.001), but this trend was not observed between PD-1^+^ and PD-1^−^ Th1 cells in HCs (9.66 ± 10.0% vs 10.1 ± 7.35%, *p* = 0.67). In addition, the frequency of SLAMF7^+^ population was significantly higher in PD-1^+^ than PD-1^−^ cTfh1 cells in IgG4-RD patients (14.0 ± 5.59% vs 0.81 ± 0.56%, *p* < 0.001), suggesting that SLAMF7^+^ cells are enriched in activated cTfh1 subsets (Fig. 3a). Again, this trend was not obvious between PD-1^+^ than PD-1^−^ cTfh1 cells in HCs (6.7 ± 2.02% vs 3.22 ± 2.92%, *p* = 0.07). Although SLAMF7^+^ fraction of Tfh1 and Th1 cells are both largely PD-1 positive, expression levels of PD-1 were significantly higher in cTfh1 cells than Th1 cells in IgG4-RD patients (Fig. 3b). Moreover, in these patients, the frequency of SLAMF7^+^ population was significantly higher in CD28^−^ than CD28^+^ Th1 cells (29.8 ± 19.2% vs 14.6 ± 11.6%, *p* = 0.01), whereas that was significantly higher in CD28^+^ than CD28^−^ cTfh1 cells (12.0 ± 4.65 vs 0.81 ± 0.77%, *p* < 0.001) (Fig. 3c). On the other hand, the frequency of SLAMF7^+^ population was not significantly higher in CD28^−^ than CD28^+^ Th1 in HCs (12.5 ± 9.57% vs 7.25 ± 3.76%, *p* = 0.36), but that was slightly higher in CD28^+^ than CD28^−^ cTfh1 cells in HCs (7.36 ± 3.34% vs 2.56 ± 3.38%, *p* = 0.04). These results suggest that SLAMF7^+^ Th1 cells are CD28^−^ Th1 cells, whereas SLAMF7^+^ cTfh1 cells are PD-1^+^CD28^+^ cTfh1 cells.

### SLAMF7^+^ activated cTfh1 as well as activated Tfh2 cells correlate with serum IgG4 levels in IgG4-RD

**Fig. 4:**
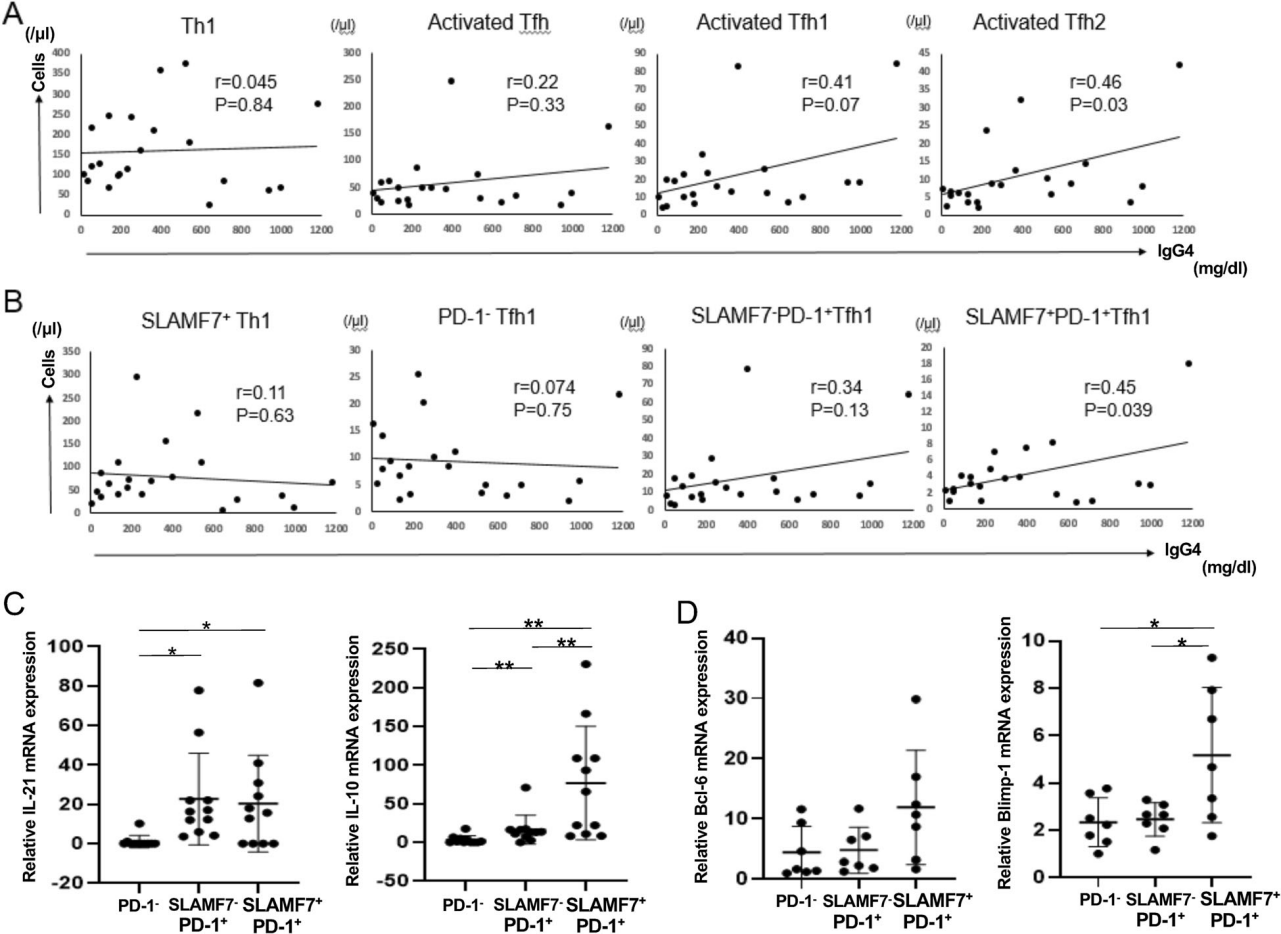
Correlation of SLAMF7^+^ activated cTfh1 cells with serum IgG4 titer and expression of IL-21 and IL-10 mRNA in Tfh1 subsets in IgG4-RD. **a** Correlation of serum titers of IgG4 with the number of Th1, activated Tfh, activated Tfh1 and activated Tfh2 cells in IgG4-RD patients. **b** Correlation of serum titers of IgG4 with the number of SLAMF7^+^ Th1, PD-1^−^ Tfh1, SLAMF7^−^PD-1^+^ Tfh1 and SLAMF7^+^PD-1^+^ Tfh1 cells in IgG4-RD patients. **c** Comparison of levels of IL-21 and IL-10 mRNA in PD-1^−^ Tfh1 cells, SLAMF7^−^PD-1^+^ Tfh1 cells and SLAMF7^+^PD-1^+^ Tfh1 cells in the peripheral blood of patients with IgG4-RD (*n* = 11). **d** Comparison of levels of Bcl-6 and Blimp-1 mRNA in PD-1^−^ Tfh1 cells, SLAMF7^−^PD-1^+^ Tfh1 cells and SLAMF7^+^PD-1^+^ Tfh1 cells in patients with IgG4-RD (*n* = 7). * indicates *p* < 0.05, ** indicates *p* < 0.01

We determined a relationship of the number of Th1 and Tfh subsets with serum IgG4 levels in IgG4-RD patients. Consistent with the previous reports [7, 8], the number of activated cTfh2 cells correlated with the titer of serum IgG4.

(*p* = 0.03, r = 0.46), whereas the number of activated cTfh1 cells and Th1 cells did not correlate with it (*p* = 0.07, r = 0.41; *p* = 0.84, r = 0.045, respectively) (Fig. 4a). Intriguingly, however, the number of SLAMF7^+^ activated cTfh1 cells correlated well with the titer of serum IgG4 (*p* = 0.039, r = 0.45), whereas the number of SLAMF7^+^ Th1 cells, PD-1^−^ cTfh1 cells, and SLAMF7^−^PD-1^+^ Tfh1 cells did not correlate with it (*p* = 0.63, r = 0.11; *p* = 0.75, r = 0.074; *p* = 0.13, r = 0.34, respectively) (Fig. 4b). To find a possible explanation for the correlation between IgG4 production and the number of SLAMF7^+^ activated cTfh1 cells, we investigated transcript levels of IL-21 and IL-10 in cTfh1 subsets in patients with IgG4-RD since IL-10 as well as IL-21 plays a critical role in plasma cell differentiation [16, 17]. As compared with non-activated (PD-1^−^) cTfh1 cells, activated (PD-1^+^) cTfh1 cells exhibited high levels of IL-21 mRNA expression irrespective of SLAMF7 expression (Fig. 4c left panel). On the other hand, the levels of IL-10 mRNA expression were significantly high in the SLAMF7^+^ fraction of activated (PD-1^+^) cTfh1 cells (Fig. 4c right panel). We also investigated transcript levels of transcription factors in cTfh1 subsets in patients with IgG4-RD. Consistent with a previous study [18], cTfh cells exhibit low levels of Bcl-6 expression, which was not affected by SLAMF7 expression (Fig. 4d left panel). On the other hand, Blimp-1 was expressed in SLAMF7^+^ Tfh1 cells at high levels, which may relate to IL-10 expression because the same is true in Treg cells [19]. Intriguingly, the levels of IL-21 mRNA expression in SLAMF7^+^ PD-1^+^ cTfh1 cells were as high as those in PD-1^+^ cTfh2 cells, whereas the levels of IL-10 mRNA expression were higher in SLAMF7^+^ PD-1^+^ cTfh1 cells than PD-1^+^ cTfh2 cells (Fig. S1). Achaete-scute homologue 2 (Ascl2), a basic helix-loop-helix transcription factor, plays a pivotal role in the initiation of Tfh cell development via upregulating CXCR5 expession as well as inhibiting Th1, Th2, and Th17 cell differentiation [20]. Intriguingly, the levels of Ascl2 mRNA expression in SLAMF7^+^PD-1^+^ cTfh1 cells were significantly lower than those in SLAMF7^−^PD-1^+^ cTfh1 cells (Fig. S2A), suggesting that Ascl2 is less involved in the function of SLAMF7^+^PD-1^+^ cTfh1 cells. Morever, CXCR5 expression in SLAMF7^+^PD-1^+^ cTfh1 cells was lower than that in SLAMF7^−^PD-1^+^ cTfh1 cells (Fig. S2B). These results suggest that SLAMF7^+^ activated cTfh1 cells have high potential to induce IgG4 secretion by B cells possibly via provision of IL-10 as well as IL-21.

### Tfh1-associated cytokines, IL-21 and IFN-γ, enhance SLAMF7 expression in memory B cells

**Fig. 5:**
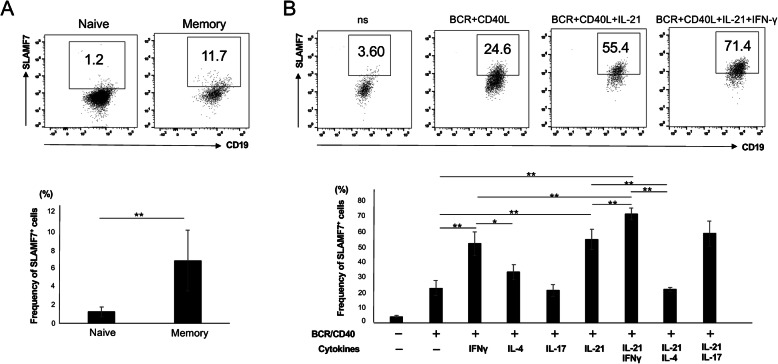
High levels of SLAMF7 expression in memory B cells in IgG4-RD and induction of SLAMF7 in memory B cells by Th-associated cytokines. **a** Comparison of SLAMF7 expression levels in naïve (CD19^+^CD20^+^IgD^+^CD27^−^CD38^−^) and memory (CD19^+^CD20^+^CD27^+^CD38^−^) cells in IgG4-RD patients (*n* = 13). **b** Purified memory B cells were cultured with Th-associated cytokines IFN-γ, IL-4, IL-17 and IL-21 along with anti-BCR and CD40 ligand for 72 h. Cells were harvested, then expression levels of SLAMF7 were evaluated by FACS analysis (*n* = 3)

Tfh cells activate antigen-specific B cells via contact help and the action of Tfh-associated cytokines. Given that SLAMF7 is a self-ligand and homotypic [12, 21, 22], we hypothesized that B cells in patients with IgG4-RD also express SLAMF7 that is involved in direct T-B interactions. To test this, we investigated the expression levels of SLAMF7 in naïve B cells, memory B cells and plasmablasts in patients with IgG4-RD. As shown in Fig. 5a, the levels of SLAMF7 expression in memory B cells were higher than those in naïve B cells, whereas almost all plasmablasts expressed SLAMF7 (data not shown). To further investigate the underlying mechanism of how SLAMF7 expression is induced in memory B cells, we tested what Th-associated cytokines are most critical for SLAMF7 expression in memory B cells. As shown in Fig. 5b, among Th-associated cytokines, IFN-γ and IL-21 significantly enhanced SLAMF7 expression in memory B cells upon stimulation via BCR and CD40, whereas IL-4 did not. Taken together, these results suggest that in addition to their cytokines for plasma cell differentiation, SLAMF7^+^ activated cTfh1 cells efficiently help memory B cells to secrete IgG4 possibly by reinforcing direct cell-to-cell interactions via SLAMF7.

## Discussion

We here demonstrate a relationship between Th subsets and SLAMF7^+^CD4^+^ T cells, both of which were previously shown to be involved in the pathogenesis of IgG4-RD. SLAMF7^+^ cells were almost exclusively observed in the activated fraction (PD1^+^) of Th1 and cTfh1 cells in patients with IgG4-RD. These SLAMF7^+^ cells, however, clearly differed in CD28 expression. Moreover, the number of SLAMF7^+^ activated cTfh1, but not Th1, cells correlated with serum IgG4 levels in IgG4-RD.

Our current findings reconcile the apparent discrepancies between previous studies in the role of cTfh1 cells in IgG4 production in IgG4-RD [7–9].

Consistent with the findings by Akiyama et al. [7, 8], the number of activated cTfh2, but not cTfh1, correlated with serum IgG4 levels (Fig. 4a). However, SLAMF7^+^ fraction of activated cTfh1 cells clearly correlated with IgG4 levels (Fig. 4b). A possible explanation for these findings is that SLAMF7^+^PD-1^+^ cTfh1 cells express high levels of IL-21 and IL-10 (Fig. 4c), both of which can promote plasma cell differentiation [16, 17]. Chen et al. showed that cTfh1 as well as cTfh2 cells in IgG4-RD efficiently promote plasma cell differentiation [9]. Our current results, thus, extend their findings by showing that activated SLAMF7^+^ cTfh1 cells play a critical role in IgG4 levels in patients.

In general, Tfh2 are efficient B-cell helper cells, whereas Tfh1 are non-efficient helpers [5]. Upon full activation, however, Tfh1 cells become an efficient helper for particularly memory B cells [23] and activated cTfh1 cells mainly contribute to the generation of high-avidity Abs following flu vaccination [24]. B cell helper functions of Tfh cells via IL-21 expression is closely associated with the levels of PD-1 expression [25]. We found that PD-1^+^ cTfh1 cells exhibited higher levels of IL-21 mRNA expression than PD-1^−^ cTfh1 cells (Fig. 4c), thus suggesting a critical marker of PD-1 levels in IL-21 production in T cells. Intriguingly, a recently identified PD-1^hi^ CXCR5^−^ T cell subset (referred to as Tph: T peripheral helper cells) has B cell helper functions via IL-21 production [26].

**Fig. 6:**
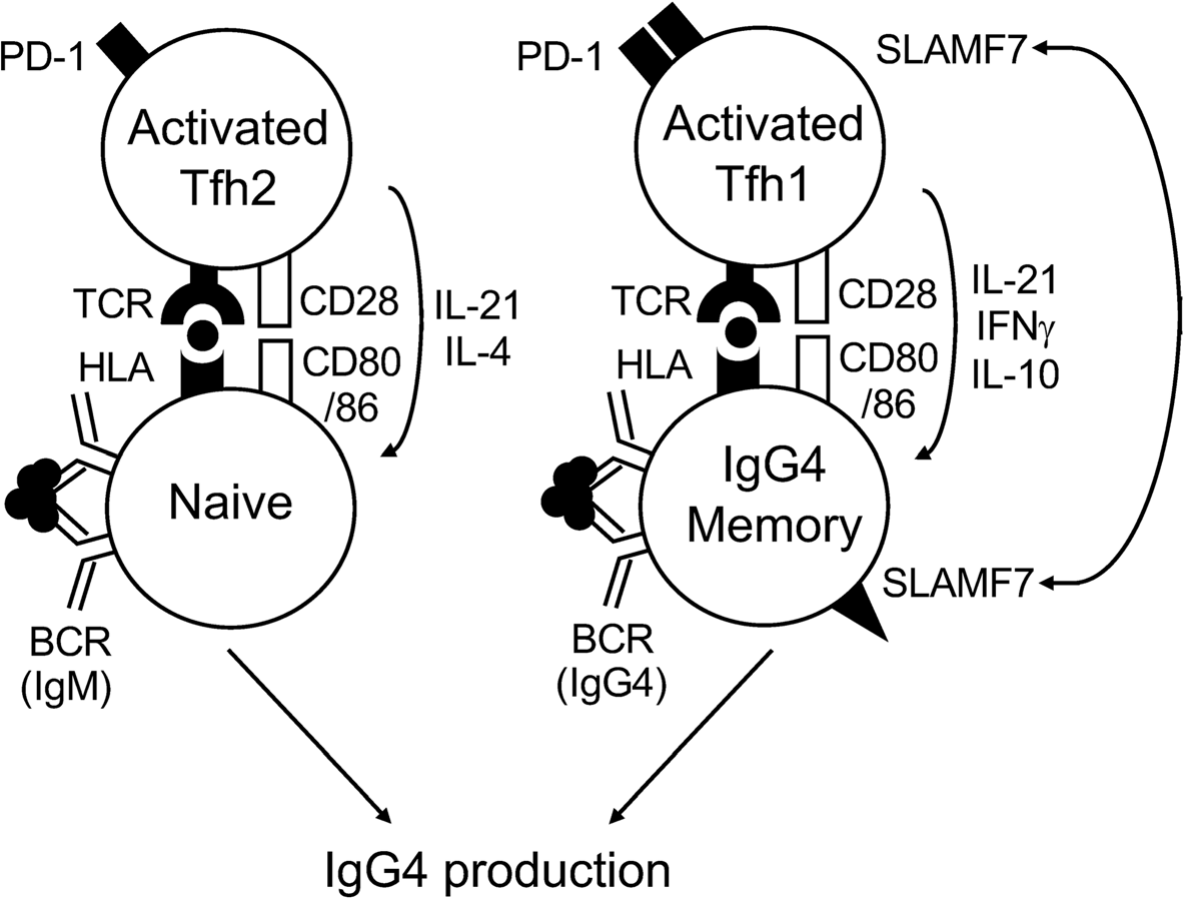
The hypothetical model in this study. Activated Tfh2 cells promote IgG4 production via interacting with naïve B cells, whereas SLAMF7^+^ activated Tfh1 cells positive for PD-1 and CD28 do so via interacting with IgG4-memory B cells. Homotypic engagement of SLAMF7 between T and B cells is also depicted. Tfh: follicular helper CD4^+^ T; SLAMF7: Signaling Lymphocyte Activation Molecule Family member 7; PD-1: Programmed cell death 1; HLA: Human Leukocyte Antigen; BCR: B-cell receptor; TCR: T-cell receptor

Our current hypothetical models are depicted in Fig. 6. Given that IL-4 and IL-21 are both required for IgG4 and IgE class switch [27, 28], activated Tfh2 cells might play a critical role in IgG4 production by naïve B cells. On the other hand, once IgG4^+^ memory B cells are generated via the GC reaction, the provision of IL-21 and IL-10 by SLAMF7^+^ activated Tfh1 cells might be sufficient for the generation of IgG4-secreting plasma cells. Indeed, the number of IgG4^+^ memory B cells is significantly increased in PB from patients with IgG4-RD than HC [29], implying the possibility that SLAMF7^+^ activated Tfh1 cells efficiently help IgG4^+^ memory B cells to differentiate into IgG4-secreting plasma cells by the mechanism mentioned above. We found that SLAMF7^+^ activated cTfh1 cells almost exclusively express CD28 (Fig. 3c). Given that CD28 stimulation is required for B cell responses to T-dependent antigens [30], CD28 expression in SLAMF7^+^ activated cTfh1 cells could further facilitate close interactions with IgG4^+^ memory B cells, leading to IgG4 production.

The SLAM family consists of nine members and is broadly expressed in hematopoietic cells [31]. In general, SLAMF7 is expressed on immune cells with cytotoxic properties such as NK cells and CD8^+^ T cells. SLAMF7-expressing cells thus express cytolytic enzymes (granzymes and perforin) and produce cytokines (TNF-α and IFN-γ) [32]. In addition to NK cells and CD8^+^ T cells, recent studies including ours showed that the subpopulation of CD4^+^ T cells also expresses SLAMF7 and has potential to produce a variety of inflammatory molecules including IL-1β, TGF-β, IFN-γ, granzyme, and perforin [14, 15]. We here found that the frequency of SLAMF7^+^ fraction is highest in Th1 cells among Th subsets (Fig. 2a). As shown in SLAMF7^+^ CD4^+^ CTLs [14], SLAMF7^+^ Th1 cells lost CD28 expression (Fig. 3c). Intriguingly, B cell depletion leads to decrease in the number of SLAMF7^+^ CD4^+^ CTLs in IgG4-RD [14], suggesting that the generation of this CTL is required for the help by B or plasmablasts. It is thus likely that in addition to IgG4 secretion, B cells exert Ab-independent functions such as antigen presentation towards T cells (Fig. 6). Indeed, this idea is well supported by the recent genome-wide association studies (GWAS) showing a significant association with *HLA-DRB1* locus in patients with IgG4-RD [33].

In addition to NK and T cells, plasma cells can also express SLAMF7. We here found that almost all plasmablasts are positive for SLAMF7. SLAMF7 is highly expressed in almost all patients with multiple myeloma, a malignant disease of plasma cells [34]. Consistent with these findings, Blimp-1, a master transcription factor for plasma cell differentiation, plays a pivotal role in SLAMF7 expression [11]. In contrast to plasma cells, there is a relative paucity of a role of SLAMF7 in B cells. A previous study showed that CD40 stimulation enhances SLAMF7 expression in human B cells [35]. We here found that SLAMF7 expression was more expressed in memory than naïve B cells in IgG4-RD (Fig. 5a). Moreover, Tfh1-associated cytokines IL-21 and IFN-γ significantly enhanced SLAMF7 expression in memory B cells stimulated via BCR and CD40 (Fig. 5b). Since SLAM family receptors have a unique property in that they are self-ligands and homotypic, we hypothesize that homotypic engagement of SLAMF7 ensures close contact between T and B cells (Fig. 6).

What then can SLAMF7 activate intracellular signaling cascades to exert functions of T and B cells? The cytoplasmic domain of SLAM family receptors generally contains one to four immunoreceptor tyrosine-based switch motifs (ITSMs). Upon SLAM engagement, the ITSM recruits its adaptor molecules SAP or EAT-2 to propagate downstream signaling, however T and B cells only express SAP but not EAT-2 [31]. Notably, SLAMF7 is only capable of binding EAT-2 but not SAP [36]. Although previous studies showed SLAMF7 engagement induces the proliferative response of CD8^+^ T cells and B cells [31, 34], an impact of SLAMF7 interactions on the function of T and B cells still remains to be elucidated.

Glucocorticoids are the mainstay treatment for IgG4-RD, however their long-term use is problematic in a disease that frequently affects middle-aged to elderly individuals [37]. Hence, there are still unmet needs in the management of this disease. Our current findings suggest that circulating SLAMF7^+^ Tfh1 cells, along with Tfh2 cells, play a pathologic role in IgG4 production in IgG4-RD. Given the possibility that SLAMF7^+^ Tfh1 can particularly support the differentiation of IgG4+ memory B cells in IgG4-RD, selective depletion of this SLAMF7^+^ subset is of potentially interest. Further understanding of this enigmatic entity will pave the avenue towards more effective treatment strategies in the future.

## Conclusions

This study has uncovered a relationship between helper CD4^+^ T (Th), particularly Tfh, cells and SLAMF7^+^ CD4^+^ T cells in IgG4-RD. Th1 cells, activated circulating Tfh1 (cTfh1), and activated cTfh2 cells increased in IgG4-RD. SLAMF7 was mainly expressed on Th1 and cTfh1, but not cTfh2, cells in the patients. Positive correlations were noted between serum IgG4 levels and the number of activated fraction of cTfh2 cells and SLAMF7^+^ cTfh1 cells, but not SLAMF7^+^ Th1 cells. Notably, activated SLAMF7^+^ cTfh1 cells were high producers of IL-10 as well as IL-21 along with high levels of Blimp-1 expression.

The frequency of SLAMF7^+^ fraction was higher in memory B cells than naïve B cells in IgG4-RD. Upon stimulation, Tfh1-associated cytokines, IL-21 and IFN-γ, most significantly induced SLAMF7 expression in memory B cells. These results suggest that circulating SLAMF7^+^ Tfh1 cells, along with Tfh2 cells, play a pathologic role in IgG4 production in IgG4-RD. Selective depletion of those populations might be more effective treatment strategies in the future.

## Methods

### Patients

We studied 21 Japanese patients with IgG4-RD at the Kyushu University hospital and 10 healthy controls (HCs). The patients fulfilled the classification criteria for IgG4-RD [38] and their clinical characteristics are shown in Table S1. All samples from patients were collected following written informed consent according to local ethics policy guidelines and the Declaration of Helsinki. We obtained the information from the medical records of the patients, including demographic data, clinical manifestations, laboratory findings and medications.

### Detection of Tfh cells, Th cells and their subsets by flow cytometry

PB mononuclear cells (PBMCs) were stained with mouse or rabbit monoclonal antibody (mAb) against human CD3, CD4, PD-1, CXCR3, CXCR5, CCR6, CD19, CD20, CD27, CD38, IgD and CD319 (SLAMF7) (all from BioLegend, San Diego, CA, USA). Circulating Tfh cells were defined as CD3^+^CD4^+^CXCR5^+^ cells and Th cells (with exclusion of Tfh cells) as CD3^+^CD4^+^CXCR5^−^ cells [5]. Tfh1, Tfh2 and Tfh17 cells were defined as CXCR3^+^CCR6^−^ cells, CXCR3^−^CCR6^−^ cells and CXCR3^−^CCR6^+^ cells among Tfh cells [5]. Th1, Th2, and Th17 were defined as CXCR3^+^CCR6^−^ cells, CXCR3^−^CCR6^−^ cells, CXCR3^−^CCR6^+^ cells among Th cells [5]. Activated Tfh cells were defined as PD-1^+^ cells among Tfh cells [5]. Naïve B cells, memory B cells and plasmablasts were defined as CD19^+^CD20^+^IgD^+^CD27^−^CD38^−^ cells, CD19^+^CD20^+^CD27^+^CD38^−^ cells and CD19^+^CD20^−^CD38^high^ cells, respectively. All samples were analysed with a FACS Aria III (BD Biosciences), and data were analyzed with FlowJo v.7.6.4 Software (Tree Star, Stanford University, CA, USA).

### Isolation and cell sorting of Tfh1 cell subsets and memory B cells

CD4^+^ T cells and CD19^+^ B cells were isolated by positive selection with CD4^+^ and CD19^+^ mAbs and a MACS magnetic cell sorting system (Miltenyi Biotec, Bergisch Gladbach, Germany). Isolated CD4^+^ T cells and CD19^+^ B cells exhibited greater than 99.5% viability and more than 95% purity, confirmed by flow cytometry. Tfh1 subsets and memory B cells were further purified by flow cytometry.

### Reagents

An affiniPure F (ab’) Fragment Goat Anti-Human IgA/ IgG/IgM (H + L) (anti-B-cell receptor [BCR], used at 10 μg/ml) was purchased from Jackson ImmunoResearch (West Grove, PA, USA). Recombinant human CD40 ligand (used at 100 ng/ml), recombinant human cytokines IFN-γ (used at 20 ng/ml), IL-4 (used at 20 ng/ml), IL-17 (used at 100 ng/ml) and IL-21 (used at 50 ng/ml)) were all from R&D Systems (Minneapolis, MN, USA).

### Quantitative real-time polymerase chain reaction (PCR)

Total RNA was extracted from purified each Tfh1 population using Isogen II reagent (Nippon Gene, Tokyo, Japan). Quantitative real-time PCR was performed in the MX3000P Sequence Detector (Agilent technologies, Santa Clara, CA, USA). The reactions were performed in triplicate wells in 96 well plates. TaqMan target mixes for *IL21* (Hs00222327_m1), *IL10* (Hs00961622_m1), *BCL6* (Hs00277037_m1)*, PRDM1* (Hs00153357_m1), and *ASCL2* (Hs00270888_s1) were all purchased from Applied Biosystems. 18S ribosomal RNA was separately amplified in the same plate as an internal control for variation in the amount of cDNA in PCR. The collected data were analyzed using Sequence Detector software (MX3000P). Data were expressed as the fold change in gene expression relative to the expression in control cells.

### Statistical analysis

Continuous variables are shown as mean ± standard error of the mean (SEM). Multiple group comparisons were analyzed using the Kruskal-Wallis test. The significance of the differences was determined by Student’s *T*-test or nonparametric Mann-Whitney *U*-test according to distributions for comparing differences between two groups. The correlations between two groups were analyzed using Spearman’s correlation coefficient. For all tests, *P*-values less than 0.05 were considered significant. All analyses were performed using JMP statistical software (SAS Institute, Cary, NC).

## Supplementary information

**Additional file 1: Figure S1.** Expression of IL-21 and IL-10 mRNA in SLAMF7^+^ activated Tfh1 cells and activated Tfh2 cells in IgG4-RD. Comparison of levels of IL-21 and IL-10 mRNA in SLAMF7^+^PD-1^+^ Tfh1 cells and PD-1^+^ Tfh2 cells in the peripheral blood of patients with IgG4-RD (*n* = 5). * indicates *p* < 0.05 (PDF format)

**Additional file 2: Figure S2.** Expression of Ascl2 mRNA and CXCR5 protein in Tfh1 subsets in patients with IgG4-RD. (A) Comparison of levels of Ascl2 mRNA in PD-1^−^ Tfh1 cells, SLAMF7^−^PD-1^+^ Tfh1 cells and SLAMF7^+^PD-1^+^ Tfh1 cells in the peripheral blood of patients with IgG4-RD (*n* = 8). (B) Comparison of surface CXCR5 expression in PD-1^−^ Tfh1 cells, SLAMF7^−^PD-1^+^ Tfh1 cells and SLAMF7^+^PD-1^+^ Tfh1 cells in IgG4-RD patients (shadow: isotype control; dotted line: SLAMF7^+^PD-1^+^Tfh1 cells; solid line: SLAMF7^−^PD-1^+^Tfh1 cells). * indicates *p* < 0.05, ** indicates *p* < 0.01. (PDF format)

**Additional file 3: Table S1.** Clinical, biological and pathological characteristics of patients with IgG4-RD and HCs. Baseline caharacteristics of patients with IgG4-RD (*n* = 21) and HC (*n* = 10) are shown. PSL: prednisolone. (PDF format)

## Data Availability

All data obtained or analyzed in this study are included in this manuscript and its supplementary information file.

## Abbreviations

SLAMF7: Signaling lymphocytic activation molecule family member 7
IgG4: Immunoglobulin G4;
IgG4-RD: Immunoglobulin G4-related disease
Tfh: T follicular helper
Th1: T helper type1
Th2: T helper type2
Th17: T helper type 17
GC: Germinal center
CXCR: CXC chemokine receptor
IL: Interleukin
cTfh: Circulating Tfh
NK cells: Natural killer cells
CTL: Cytotoxic T lymphocytes
PB: Plasmablast
HCs: Healthy controls
PBMCs: Peripheral blood mononuclear cells
PD-1: Programmed cell death 1
CCR: C-C chemokine receptor
FACS: Fluorescence activated cell sorter
mAb: Monoclonal antibody
MACS: Magnetic activated cell sorting
BCR: B cell receptor
CD40L: CD40 Ligand
IFN: Interferon
PCR: Polymerase chain reaction
RNA: Ribonucleic acid
DNA: Deoxyribonucleic acid
cDNA: Complementary DNA
mRNA: Messenger RNA
IgE: Immunoglobulin E
TGF: Transforming growth factor
GWAS: Genome-wide association studies
HLA: Human leukocyte antigen
ITSMs: Immunoreceptor tyrosine-based switch motifs
SAP: Signaling lymphocyte activation molecule-associated protein
EAT-2: EWS/Fli1 activated transcript 2
SEM: Standard error of the mean
SD: Significance of differences
TCR: T cell receptor
Ascl2: Achaete-scute homologue 2
