# Supplementary figures and images for: Association of circulating SLAMF7+Tfh1 cells with IgG4 levels in patients with IgG4-related disease
Source: BMC Immunol. 2020 Jun 1;21:31. doi: 10.1186/s12865-020-00361-0 (PMC7268355; doi:10.1186/s12865-020-00361-0)

Figure S1

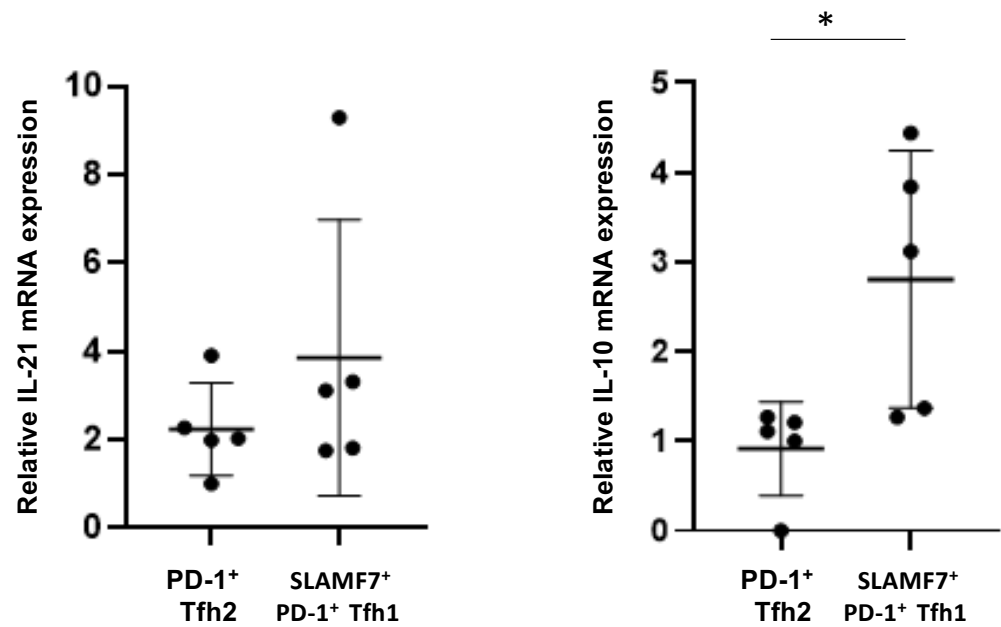

Supplement: Supplementary file 1 — Additional file 1: Figure S1. Expression of IL-21 and IL-10 mRNA in SLAMF7+ activated Tfh1 cells and activated Tfh2 cells in IgG4-RD. Comparison of levels of IL-21 and IL-10 mRNA in SLAMF7+PD-1+ Tfh1 cells and PD-1+ Tfh2 cells in the peripheral blood of patients with IgG4-RD (n = 5). * indicates p < 0.05 (PDF format) [file 12865_2020_361_MOESM1_ESM.pdf]

Figure S2

A

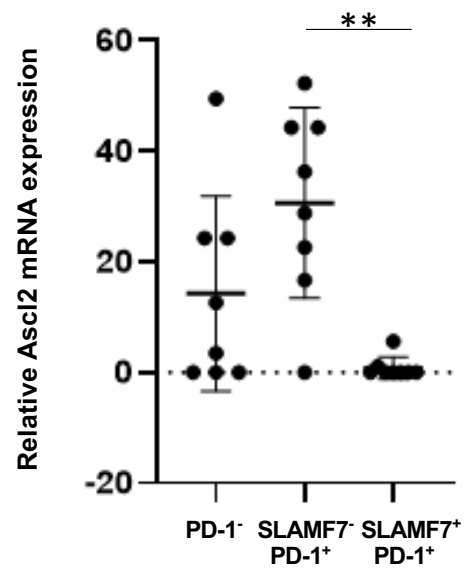

B

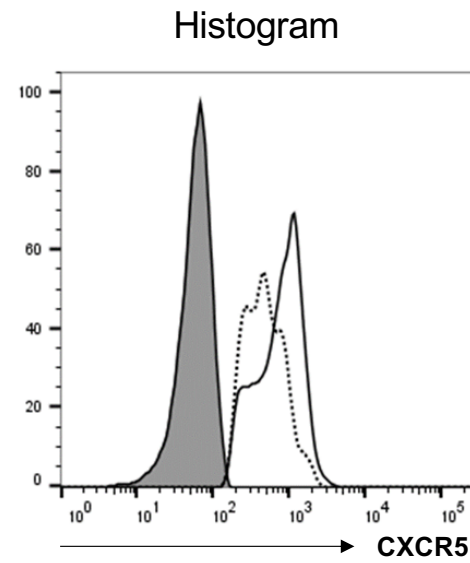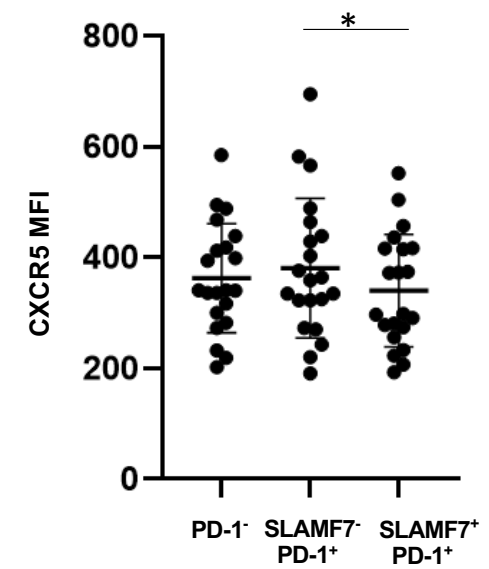

Supplement: Supplementary file 2 — Additional file 2: Figure S2. Expression of Ascl2 mRNA and CXCR5 protein in Tfh1 subsets in patients with IgG4-RD. (A) Comparison of levels of Ascl2 mRNA in PD-1− Tfh1 cells, SLAMF7−PD-1+ Tfh1 cells and SLAMF7+PD-1+ Tfh1 cells in the peripheral blood of patients with IgG4-RD (n = 8). (B) Comparison of surface CXCR5 expression in PD-1− Tfh1 cells, SLAMF7−PD-1+ Tfh1 cells and SLAMF7+PD-1+ Tfh1 cells in IgG4-RD patients (shadow: isotype control; dotted line: SLAMF7+PD-1+Tfh1 cells; solid line: SLAMF7−PD-1+Tfh1 cells). * indicates p < 0.05, ** indicates p < 0.01. (PDF format) [file 12865_2020_361_MOESM2_ESM.pdf]
