# Supplementary material for: Association of circulating SLAMF7+Tfh1 cells with IgG4 levels in patients with IgG4-related disease
Source: BMC Immunol. 2020 Jun 1;21:31. doi: 10.1186/s12865-020-00361-0 (PMC7268355; doi:10.1186/s12865-020-00361-0)
Supplement: Supplementary file 3 — Additional file 3: Table S1. Clinical, biological and pathological characteristics of patients with IgG4-RD and HCs. Baseline caharacteristics of patients with IgG4-RD (n = 21) and HC (n = 10) are shown. PSL: prednisolone. (PDF format) [file 12865_2020_361_MOESM3_ESM.pdf]

Table S1

	Patients with IgG4-RD	Healthy controls
Male, n (%)	15 (71)	6 (60)
Age (years), average (SD)	66 (12.4)	52 (10.9)
Serum IgG4 (mg/dl), average (SD)	372 (336)	37 (21)
Serum IgG (mg/dl), average (SD)	1645 (545)	
CRP (mg/dl), average (SD)	0.3 (0.67)	
Hypocomplement, n (%)	3 (14)	
Untreated patients, n (%)	13 (62)	
Drug usage, (n)	PSL (8)	
Dose of PSL (mg/day), average (SD)	6.05 (2.83)	
SS-A positive, n (%)	0 (0)	
Organs involved		
Salivary gland, n (%)	17 (81.0)	
Lymph node, n (%)	7 (33.3)	
Pancreas, n (%)	6 (28.6)	
Peritoneum, n (%)	6 (28.6)	
Lung, n (%)	5 (23.8)	
Lacrimal gland, n (%)	4 (19.0)	
Prostate, n (%)	3 (14.3)	
Aorta, n (%)	3 (14.3)	
Kidney, n (%)	3 (14.3)	
Thyroid, n (%)	1 (4.8)	
